# DnaJB6 is a RanGTP-regulated protein required for microtubule organization during mitosis

**DOI:** 10.1242/jcs.227033

**Published:** 2019-06-03

**Authors:** Miquel Rosas-Salvans, Jacopo Scrofani, Aitor Modol, Isabelle Vernos

**Affiliations:** 1 Centre for Genomic Regulation (CRG), Barcelona Institute of Science and Technology, Dr Aiguader 88, 08003 Barcelona, Spain; 2 Universitat Pompeu Fabra (UPF), 08003, Barcelona, Spain; 3 ICREA, Passeig de Lluis Companys 23, 08010 Barcelona, Spain

**Keywords:** DnaJB6, RanGTP, Spindle organization, Dynein, Microtubules

## Abstract

Bipolar spindle organization is essential for the faithful segregation of chromosomes during cell division. This organization relies on the collective activities of motor proteins. The minus-end-directed dynein motor complex generates spindle inward forces and plays a major role in spindle pole focusing. The dynactin complex regulates many dynein functions, increasing its processivity and force production. Here, we show that DnaJB6 is a novel RanGTP-regulated protein. It interacts with the dynactin subunit p150Glued (also known as DCTN1) in a RanGTP-dependent manner specifically in M-phase, and promotes spindle pole focusing and dynein force generation. Our data suggest a novel mechanism by which RanGTP regulates dynein activity during M-phase.

## Abstract (precis)

**Summary:** DnaJB6 is a novel RanGTP-regulated protein that appears to play an important role in dynein-dependent spindle organization and spindle assembly.

## INTRODUCTION

Spindle assembly involves the organization of microtubules into two antiparallel arrays with interdigitating plus-ends, and two focused minus-ends forming the spindle poles. This organization is highly dependent on the collective activities of microtubule-dependent motor proteins. The plus-end-directed homotetrameric motor Eg5 (also known as KIF11) generates outward forces that, together with other mechanisms, separate the spindle poles (Blangy et al., 1995; Kapitein et al., 2005; Kashina et al., 1996, 1997; Whitehead and Rattner, 1998). Indeed, interfering with Eg5 activity results in the assembly of monopolar spindles with unseparated centrosomes (Mayer et al., 1999). Interestingly, impairing dynein activity rescues spindle bipolarity in Eg5-inhibited cells (Ferenz et al., 2009; Mitchison et al., 2005; Raaijmakers et al., 2013; Tanenbaum et al., 2008). This suggests that a fine balance between outward forces generated by Eg5 and inward forces generated by dynein defines spindle length and bipolarity (Ferenz et al., 2009; Florian and Mayer, 2012; Gaglio et al., 1997, 1996; Heald et al., 1996; Tanenbaum et al., 2008; Walczak et al., 1998).

In addition to force generation within the spindle, dynein is also essential for focusing microtubule minus-ends into the spindle poles (Echeverri et al., 1996; Gaglio et al., 1996; Heald et al., 1996; Mitchison et al., 2005; Tan et al., 2018; Walczak et al., 1998; Wittmann and Hyman, 1999). This process involves NuMA (also known as NUMA1), which recruits the dynein–dynactin complex to the microtubule minus-ends, promoting the transport and focusing of the microtubule minus-ends (Compton and Cleveland, 1993; Gaglio et al., 1995; Haren et al., 2009; Hueschen et al., 2017; Khodjakov et al., 2003; Kisurina-Evgenieva et al., 2004; Merdes et al., 2000, 1996; Silk et al., 2009). At the poles, dynein is also involved in centrosome coupling to the spindle. Indeed, interfering with dynein activity induces centrosome positioning defects or detachment from the spindle pole (Heald et al., 1997; Gaglio et al., 1997; Morales-Mulia and Sholey, 2005; Goshima et al., 2005). During mitosis, dynein is also recruited to the cell membrane through interaction with different protein complexes. Cortical dynein localization and dynein-dependent force generation on astral microtubules regulate spindle positioning and orientation (di Pietro et al., 2016; Lu and Johnston, 2013).

During M-phase, RanGTP triggers the nucleation, stabilization and organization of microtubules in the vicinity of the chromosomes, playing an essential role in bipolar spindle assembly (Carazo-Salas et al., 1999; Cavazza and Vernos, 2015; Clarke and Zhang, 2008). The general mechanism involves the release of a specific group of nuclear localization signal (NLS)-containing proteins from inhibitory interactions with importins (Görlich et al., 1995, 1996). Several RanGTP-regulated proteins have been identified and shown to play essential roles in spindle assembly (Cavazza and Vernos, 2015). Recently, we used a mass spectrometry-based proteomic approach to identify the full proteome associated with RanGTP-induced microtubules in *Xenopus* egg extracts and validated a few selected proteins with no reported function in mitosis (Rosas-Salvans et al., 2018). One of them, DnaJB6 (also known as MRJ, HSJ2 or MSJ1), had also been identified in mitotic spindle proteomes (Bonner et al., 2011; Rao et al., 2016; Sauer et al., 2005) although its function in mitosis had not been addressed. DnaJB6 is an heat-shock protein 40 kDa (HSP40) family protein with two alternatively spliced isoforms (in humans). These two isoforms share the first 239 amino acids but differ at their C-terminus. The short isoform (DnaJB6-S, 241 amino acids) is cytoplasmic, whereas the long isoform (DnaJB6-L, 326 amino acids) has a nuclear localization signal at its C-terminus (Mitra et al., 2008), and has been shown to localize to the nucleus (Cheng et al., 2008; Mitra et al., 2008). The co-chaperon activity of the short isoform has been demonstrated *in vitro* (Chuang et al., 2002). Additionally, DnaJB6 prevents huntingtin aggregation *in vitro* independently of its DnaJ domain (and HSP70), although *in vivo* this domain seems to be required (Chuang et al., 2002; Hageman et al., 2010). DnaJB6-L expression is associated with suppression of tumorigenesis and metastasis in breast cancer (Meng et al., 2016). Interestingly, DnaJB6 is highly expressed in human testis, ovary, liver and placenta (Seki et al., 1999), and its expression is increased during mitosis in HeLa cells (Dey et al., 2009; Seki et al., 1999). We have previously shown that DnaJB6 localizes to the spindle poles and its silencing affects microtubule aster formation in cells undergoing microtubule regrowth, suggesting that it is a novel RanGTP-regulated spindle assembly factor (SAF) (Rosas-Salvans et al., 2018).

Here, we show that DnaJB6 interacts with the dynactin complex component p150Glued (also known as DCTN1) in a RanGTP-dependent manner in pulldown experiments. Moreover, DnaJB6 silencing induces multiple spindle defects that are all consistent with defective dynein function. These data suggest that dynein could be regulated during mitosis through a novel mechanism involving DnaJB6 and RanGTP.

## RESULTS

### DnaJB6 is a RanGTP-regulated protein that localizes to the spindle

**Fig. 1.**
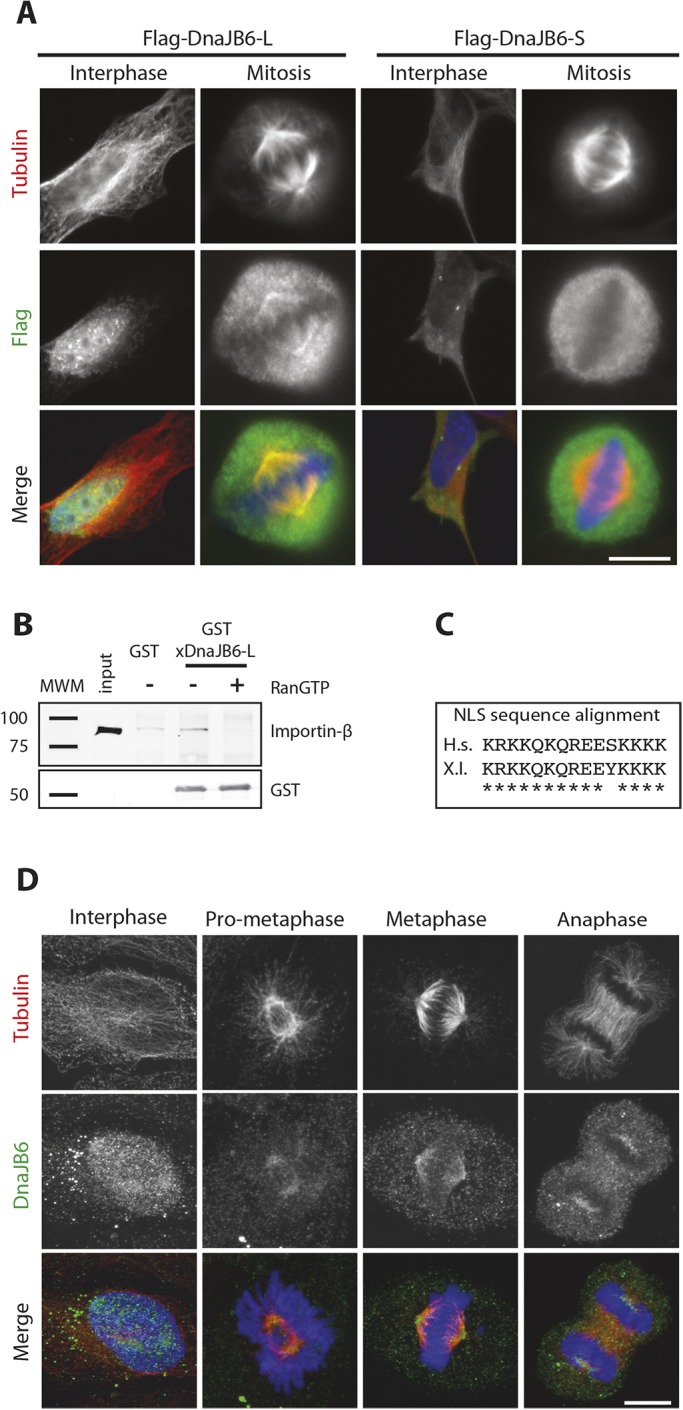
**DnaJB6 is a RanGTP-regulated protein associated to the mitotic spindle.** (A) Immunofluorescence on HeLa cells transfected with Flag-tagged long and short DnaJB6 isoform-expressing constructs. DnaJB6-L (long) localizes in the nucleus during interphase and to the spindle poles in mitosis. DnaJB6-S (short) shows diffuse cytoplasmic localization both during interphase and mitosis. (B) Western blot analysis of a GST–xDnaJB6-L pulldown experiment in *Xenopus laevis* egg extract. Importin-β associates with GST–xDnaJB6-L and is released by addition of RanGTP to the extract (top). The lower panel shows that similar levels of GST–xDnaJB6 were used for pull downs in the presence or absence of RanGTP. (C) Amino acid sequence alignment of the putative NLS in human (top) and *Xenopus laevis* (bottom) DnaJB6 long isoforms. Asterisks highlight identical amino acids. (D) Immunofluorescence images of HeLa cells showing the localization of DnaJB6 in different cell cycle stages using an in-house generated antibody. DnaJB6 accumulates in the nucleus during interphase and localizes to the spindle during mitosis with an accumulation at the spindle poles in metaphase. Tubulin is shown in red, DnaJB6 in green and DNA in blue. Scale bars: 10 μm.

DnaJB6 has two alternatively spliced isoforms in humans that differ at their C-terminus. To determine the localization of these proteins in interphase and in mitosis, we performed immunofluorescence studies in cells expressing the long or the short isoform of DnaJB6 tagged with FLAG. In agreement with previous reports, DnaJB6-S localized to the cytoplasm in interphase, whereas DnaJB6-L localized to the nucleus (Cheng et al., 2008; Mitra et al., 2008). In mitotic cells, DnaJB6-S did not show any specific localization. By contrast, DnaJB6-L localized to the spindle in metaphase cells (Fig. 1A).

We then used a *Xenopus* egg extract system to directly test whether the long isoform of DnaJB6 (DnaJB6-L; xDnaJB6-L refers to the *Xenopus* form), which contains an NLS, could be regulated by RanGTP during mitosis. GST–xDnaJB6-L-coated beads were incubated in cytostatic factor (CSF)-arrested egg extract supplemented or not with RanGTP. Western blot (WB) analysis of the proteins associated with the beads showed that importin-β was specifically pulled down by GST–xDnaJB6-L in the absence but not in the presence of RanGTP (Fig. 1B). This result suggests that DnaJB6 is regulated by RanGTP during mitosis as is seen for other SAFs (Cavazza and Vernos, 2015). The high degree of conservation of the NLS sequence of the *Xenopus* and human DnaJB6 proteins (Fig. 1C) suggests that the interaction with importin-β could be conserved in human.

We then used immunofluorescence microscopy to characterize the subcellular localization of endogenous DnaJB6 during the cell cycle, using an anti-DnaJB6 antibody that recognizes both isoforms in western blotting experiments. The anti DnaJB6 signal was predominant in the nucleus during interphase with some signal in the cytoplasm. Mitotic cells showed a clear signal for DnaJB6 on the spindle microtubules with an accumulation at the spindle poles (Fig. 1D).

### DnaJB6 has a role in microtubule organization during mitosis

**Fig. 2.**
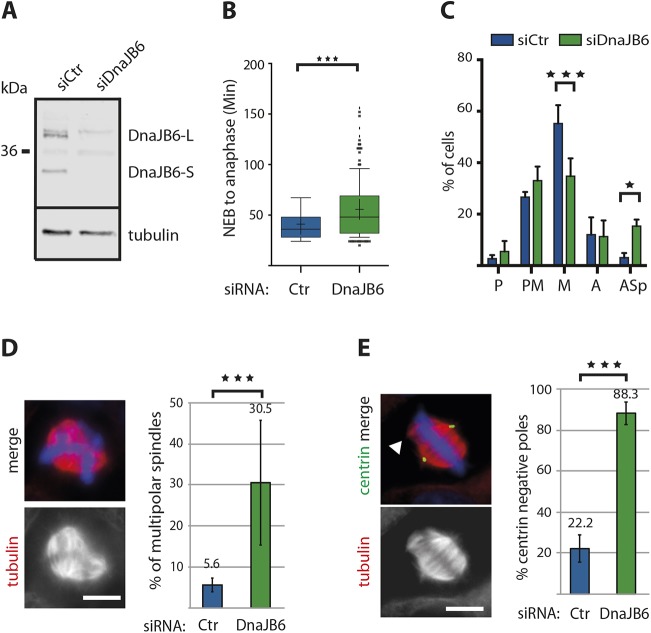
**DnaJB6 is required for bipolar** **spindle assembly.** (A) Western blot analysis of control and DnaJB6-silenced cell lysates showing the efficiency of DnaJB6 silencing. Cells were lysed 48 h post transfection and 40 μg of protein were loaded per lane. DnaJB6 silencing efficiency was 90% for DnaJB6-L and more than 90% for DnaJB6-S. Tubulin is blotted as protein loading control. (B) Box-and-whisker plot showing the time from nuclear envelope breakdown (NEB) to anaphase onset for control and DnaJB6-silenced cells. Data are from three independent experiments in which a total of 281 control and 250 DanJB6 silenced cells were analyzed. ***P<0.0001 (Mann–Whitney test). (C) Bar graph showing the mean±s.d. distribution of mitotic phases in control (blue) or DnaJB6-silenced HeLa cells (green). Prophase (P), prometaphase (PM), metaphase (M), anaphase (A) and aberrant spindles (Asp) are quantified. Monopolar, multipolar spindles and more disorganized structures were included into the aberrant spindle category (Asp). Data from three independent experiments in which a total of 454 control and 498 DnaJB6-silenced cells were analyzed. *P<0.05, ***P<0.0001 (ANOVA test). (D) Multipolar spindle are highly represented in DnaJB6-silenced cells. Left: representative image of a DnaJB6-silenced HeLa cell with a multipolar spindle. Tubulin is in red and DNA in blue. Scale bar: 10 μm. Right: quantification (mean±s.d.) of the percentage of multipolar spindles in control (blue) and DnaJB6-silenced (green) cells. The percentages are indicated on top of the columns. Data are from three independent experiments in which a total of 765 control and 778 DnaJB6-silenced cells were analyzed. ***P<0.0001 (Fisher's exact test). (E) DnaJB6-silenced cells present extra poles that are mainly not centrosomal. Left: immunofluorescence images of a DnaJB6-silenced HeLa cell with a multipolar spindle showing tubulin (red), centrin (green) and DNA (blue). The arrowhead points to a centrin negative pole. Scale bar: 10 μm. Right: quantification (mean±s.d.) of the percentage of multipolar spindles with poles negative for centrin in control (blue) and DnaJB6-silenced (green) HeLa cells. The percentages are indicated on top of the columns. Data are from two independent experiments in which a total of 126 control and 116 DnaJB6-silenced cells were analyzed. ***P<0.0001 (ANOVA test).

To determine whether DnaJB6 has any role during mitosis, we monitored mitosis progression by performing time-lapse fluorescence microscopy in control and DnaJB6-silenced HeLa cells expressing H2B–eGFP and α-tubulin–mRFP. DnaJB6 silencing efficiently reduced the levels of the protein (Fig. 2A) and induced a delay in mitosis. Indeed, DnaJB6-silenced cells needed 35.5% more time than control cells to reach anaphase onset from nuclear envelope breakdown (NEB) (Fig. 2B). To determine the causes for this delay, we examined microtubule organization and chromosome positioning in fixed control and DnaJB6-silenced HeLa cells by immunofluorescence microscopy. The quantification of the distribution of the mitotic phases showed a significant decrease in the proportion of DnaJB6-silenced cells in metaphase (Fig. 2C). Concomitantly, the percentage of cells with spindle defects increased significantly. Additionally, an increase of the percentage of pro-metaphase cells was observed, although this was not statistically significant (Fig. 2C).

**Fig. 3.**
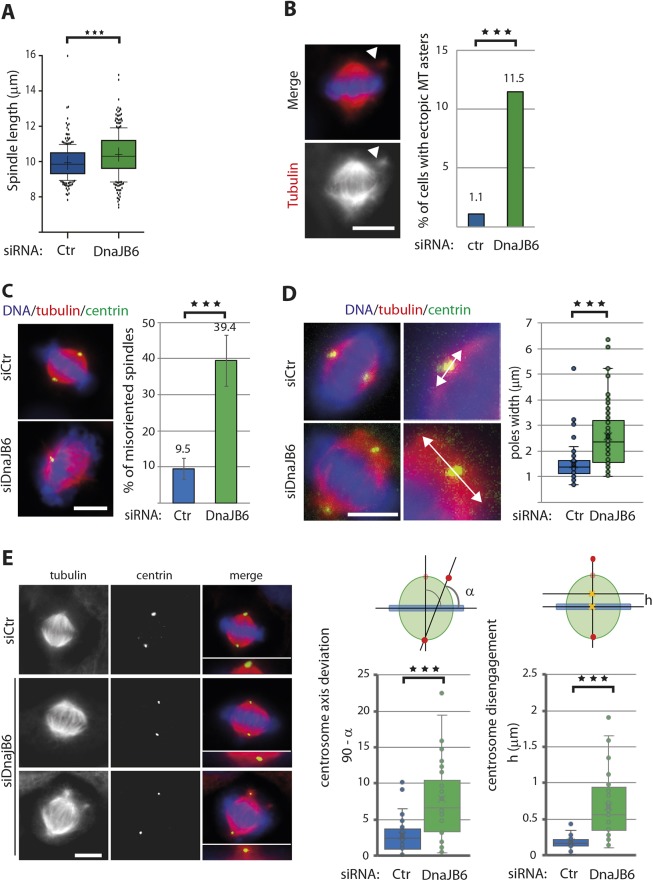
**DnaJB6 is required for spindle orientation and spindle poles organization.** (A) Box-and-whisker plot showing the spindle length in control and DnaJB6-silenced HeLa cells. The spindle length of 267 control and 294 DnaJB6-silenced cells from three independent experiments was measured. A statistically significant increase of the length was detected in DnaJB6-silenced cells. ***P<0.0001 (Mann–Whitney test). (B) DnaJB6-silenced cells have ectopic microtubule clusters in metaphase. Left: immunofluorescence images of a representative DnaJB6-silenced metaphase cell with an ectopic microtubule cluster (white arrowhead). Tubulin is shown in red and DNA in blue. Scale bar: 10 μm. Right: bar graph showing the percentage of bipolar spindles containing ectopic microtubule clusters in control and DnaJB6-silenced HeLa cells. 182 control and 192 DnaJB6-silenced cells in metaphase were examined. ***P<0.001 (Fisher's exact test). (C) DnaJB6-silenced cells present spindle orientation defects. Left: representative images of a control cell with a correctly oriented spindle in which the two centrosomes are aligned with the substrate (top image) and a DnaJB6-silenced cell with a misoriented spindle (bottom image; in this case, only one centrosome is visible due to the location of the two poles on two different focal planes). Tubulin is in red, centrin in green and DNA in blue. Scale bar: 10 μm. Right: quantification of the mean±s.d. percentage of metaphase HeLa cells that present misoriented spindles. Spindles were scored as not correctly oriented with the substrate when the two centrosomes were not on the same focal plane. Anti-centrin antibodies were used to determine centrosome position. The percentages are indicated on top of the columns. Data from four independent experiments in which a total of 600 cells were analyzed for condition. ***P<0.001 (Student's *t*-test). (D) DnaJB6-silenced cells show severe spindle pole focusing defects. Left: immunofluorescence images of control and DnaJB6-silenced HeLa cells showing tubulin (red), centrin (green) and DNA (blue). Spindles from a control and a DnaJB6-silenced are shown as examples of focused and open spindle poles, respectively. Double arrows exemplify poles width measurement. Scale bar: 10 μm. Right: box-and-whisker plot showing measures of spindle pole width in control and DnaJB6-silenced HeLa cells. Bipolar metaphase spindles with correctly aligned chromosomes were randomly selected. The tubulin signal was thresholded and a line was drawn crossing the centrosome (centrin staining) and connecting the two closest spindle borders. Graph shows the results of a representative experiment out of two in which a total of 90 poles were measured in each condition. ***P<0.001 (Student's *t*-test). (E) DnaJB6-silenced cells show centrosome mispositioning relative to the spindle pole position. Left: representative images of a control and two DnaJB6-silenced cells. Centrosomes are laterally displaced or detached from the spindle poles in DnaJB6-silenced cells. Tubulin is in red, centrin is green and DNA is blue. Scale bar: 10 μm. Right: quantification of the centrosome position defects. When the centrosomes move laterally the centrosome axis is deviated. The left-hand box-and-whisker plot represents the centrosome axis deviation in control and DnaJB6-silenced cells. This deviation is calculated by measuring the α and subtracting this from 90, where α is the angle between the centrosome axis and the metaphase plate, as shown in the schematic representation. When the centrosomes are detached from the poles a midpoint is fixed (yellow cross) and distance from the metaphase plate (h) measured and plotted (shown on the right). The plots show data from a representative experiment where more than 30 cells were analyzed for each condition. ***P<0.001 (Student's *t*-test).

One major phenotype was a significant increase of the proportion of cells with multipolar spindles in DnaJB6-silenced cells compared to controls (30.5% versus 5.6%) (Fig. 2D). This phenotype was not associated with centrosome amplification. Indeed, 88% of the multipolar spindles assembled in DnaJB6-silenced cells had only two centrin-positive poles (Fig. 2E). Upon closer examination bipolar spindles assembled in DnaJB6-silenced cells also showed a range of defects. They were significantly longer than those assembled in control cells (10.4 μm in DnaJB6-silenced cells versus 9.9 μm in control cells) (Fig. 3A) and, in some cases (11.5% in DnaJB6-silenced cells and 1.1% in control cells), small ectopic microtubule clusters were in their proximity, suggesting defective incorporation of some microtubules to the main spindle body (Fig. 3B). Overall, there was also an increase of spindles not correctly oriented with respect to the substrate in DnaJB6-silenced cells (Fig. 3C). Moreover, bipolar spindles assembled in DnaJB6-silenced cells had pole focusing defects with a significantly wider pole width than control bipolar spindles (Fig. 3D). In addition, some centrosomes were loosely attached to the spindle pole (Fig. 3E) and the centrosomes axis was altered showing a deviation with respect to the spindle axis (Fig. 3E).

Taken together, these results suggest a role for DnaJB6 in microtubule organization during mitosis.

### DnaJB6 has a direct role in microtubule organization in M-phase

**Fig. 4.**
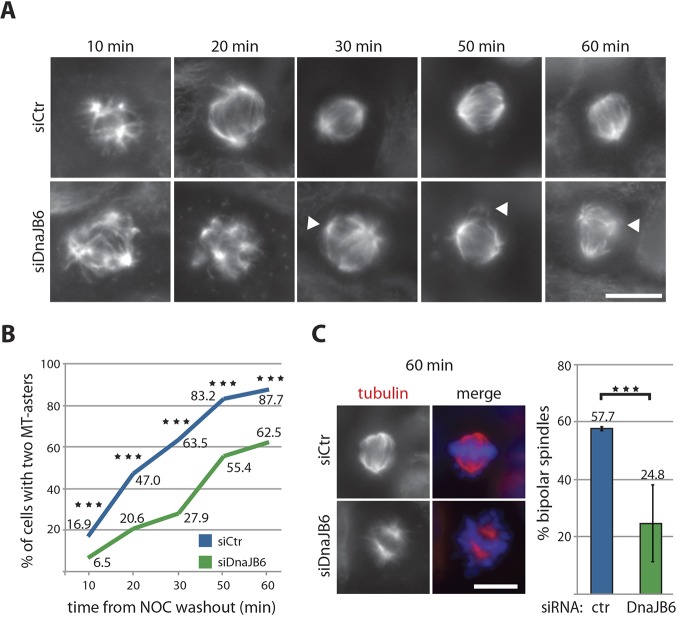
**DnaJB6 is involved in spindle microtubules organization.** (A) Immunofluorescence images of mitotic control and DnaJB6-silenced HeLa cells undergoing microtubule regrowth after nocodazole washout. Cells were fixed at the times indicated after nocodazole washout and processed for immunofluorescence to visualize the microtubules (tubulin, shown in gray) and DNA (not shown). The white arrowheads point to microtubule asters that fail to incorporate into the main spindle poles. Scale bar: 10 μm. (B) Quantification of the percentage of cells with two asters at different time points after nocodazole (NOC) washout as shown in A. The blue line corresponds to the control cells and the green line to DnaJB6-silenced cells. Data are from three independent experiments in which more than 300 cells were recorded for each time point. ***P<0.001 (Fisher's exact test). (C) Representative immunofluorescence image of a HeLa cell fixed at 60 min after nocodazole washout with two microtubule asters that do (top) or do not (bottom) organize a bipolar spindle. Tubulin is in red, and DNA in blue. Scale bar: 10 μm. Quantification of the mean±s.d. percentage of cells having two microtubule asters organized into a bipolar spindle 60 min after nocodazole washout. Data from two independent experiments in which 503 control (blue) and 638 DnaJB6-silenced cells (green) were analyzed. The exact percentages are indicated on top of each bar. ***P<0.001 (Fisher's exact test).

To gain further support for the hypothesis that DnaJB6 is involved in microtubule organization during mitosis, we monitored microtubule organization in control and DnaJB6-silenced cells undergoing regrowth after nocodazole washout. Cells were fixed at different time points and processed for immunofluorescence (Fig. 4A). The efficiency of bipolar spindle organization was measured by quantifying the number of microtubule asters at the different time points (Fig. S1). In most control cells, the number of microtubule asters was initially high due to the activity of the centrosomal and chromosomal pathways (Meunier and Vernos, 2011), and reduced over time so that finally only two microtubule asters defined the poles of the bipolar spindle. In contrast, the percentage of cells with only two microtubule asters was significantly reduced in DnaJB6-silenced cells at all the time points examined (Fig. 4B) and the difference with the control increased with time. At 60 min after nocodazole washout, 57.7% of the control cells had two asters organized into a bipolar spindle, whereas this percentage was reduced to 24.8% in DnaJB6-silenced cells (Fig. 4C). These data suggest that the role of DnaJB6 in microtubule organization is associated with microtubule aster clustering and spindle pole focusing.

**Fig. 5.**
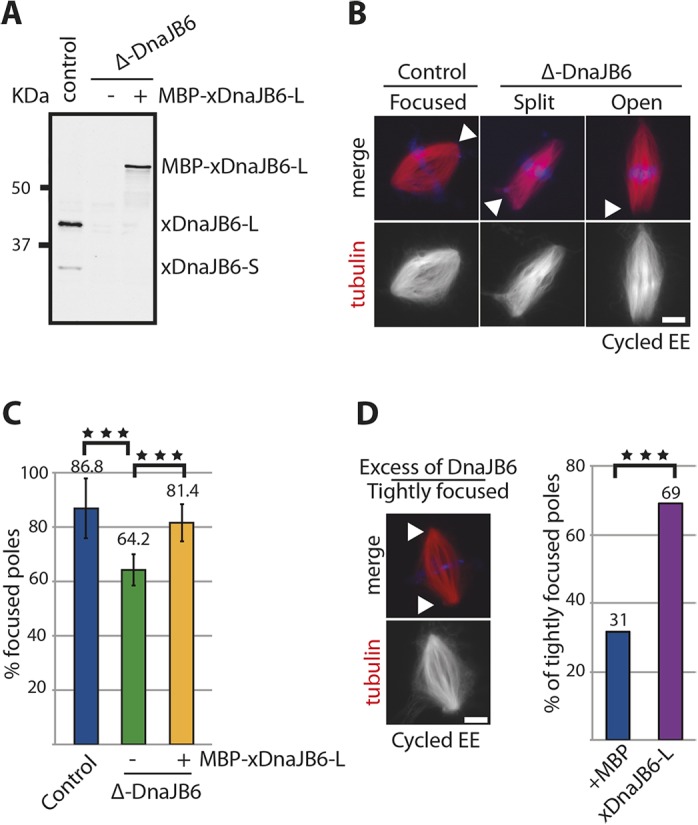
**DnaJB6 depletion from *Xenopus* egg extracts induces spindle pole organization defects.** (A) Western blot analysis of control and xDnaJB6-depleted (Δ-DnaJB6) *Xenopus* egg extracts (EE) without (−) or with (+) addition of the recombinant MBP–xDnaJB6-L. The depletion was very efficient and the recombinant protein was added at close to endogenous concentrations. 1 μl of egg extract was loaded per lane. The endogenous and recombinant proteins were detected with an in-house generated anti-xDnaJB6 antibody. (B) Fluorescence images of spindles assembled in control or DnaJB6-depleted egg extracts. Most spindles assembled in control-depleted extracts have focused spindle poles, whereas many spindles assembled in DnaJB6-depleted extracts show spindle pole focusing defects defined as ‘Split poles’ or ‘Open poles’ as shown. The white arrowheads point to the different types of spindle poles. In the merge, tubulin is shown in red and DNA in blue. Scale bar: 10 μm. (C) Graph showing the mean±s.d. percentage of focused spindle poles in control extracts (blue), DnaJB6-depleted extracts (green) and DnaJB6-depleted extracts containing MBP–xDnaJB6 (orange). Data are from four independent experiments in which 703, 607 and 358 spindle poles were examined, respectively. ***P<0.001 (ANOVA test). (D) Fluorescence image of a spindle assembled in egg extract supplemented with an excess of DnaJB6 (1 μM) showing tightly focused spindle poles (white arrowheads). Scale bar: 10 μm. Bar graph on the right shows the percentage of tightly focused poles in spindles assembled in control extracts supplemented with MBP (blue) and in extracts supplemented with MBP–xDnaJB6 (purple). Data are from three independent experiments in which 114 and 258 spindle poles were analyzed. ***P<0.0001 (Fisher exact test).

Since DnaJB6 is a member of the Hsp40 co-chaperon family, its absence could potentially lead to an accumulation of protein folding defects that could be responsible for the phenotypes that we observed in mitosis. To rule out this possibility, we used the *Xenopus* egg extract system. Cycled spindle assembly experiments were performed in control and DnaJB6-depleted egg extracts and maltose-binding protein (MBP) or recombinant MBP–xDnaJB6-L were added to the control and depleted extracts for rescue experiments (Fig. 5A). Samples were collected 60 min after cycling the extract into mitosis and processed for immunofluorescence. Although bipolar spindles assembled with similar efficiency in control and DnaJB6-depleted extracts (data not shown), the absence of DnaJB6 resulted in spindle pole defects (Fig. 5B). While 86.8% of the control spindles had focused poles, spindles assembled in DnaJB6-depleted extracts had a statistically significant reduction of focused spindle poles to 64% and a high proportion of open spindle poles, sometimes even generating two sub-poles. This phenotype was rescued by addition of MBP–xDnaJB6-L to the depleted extract promoting the formation of focused spindle poles in 81% of samples (Fig. 5C). These results showed that the spindle pole focusing defects can be entirely attributed to the absence of DnaJB6 specifically during mitosis. Consistently, the addition of an excess of MBP-xDnaJB6-L to the egg extracts as they were cycled into mitosis resulted in an increase of the percentage of very tightly focused spindle poles (Fig. 5D).

Together, these results indicate that DnaJB6 plays a direct role in microtubule focusing at the spindle poles during M-phase.

### DnaJB6 interacts with p150Glued in a RanGTP-dependent manner during mitosis and is required for its spindle localization

**Fig. 6.**
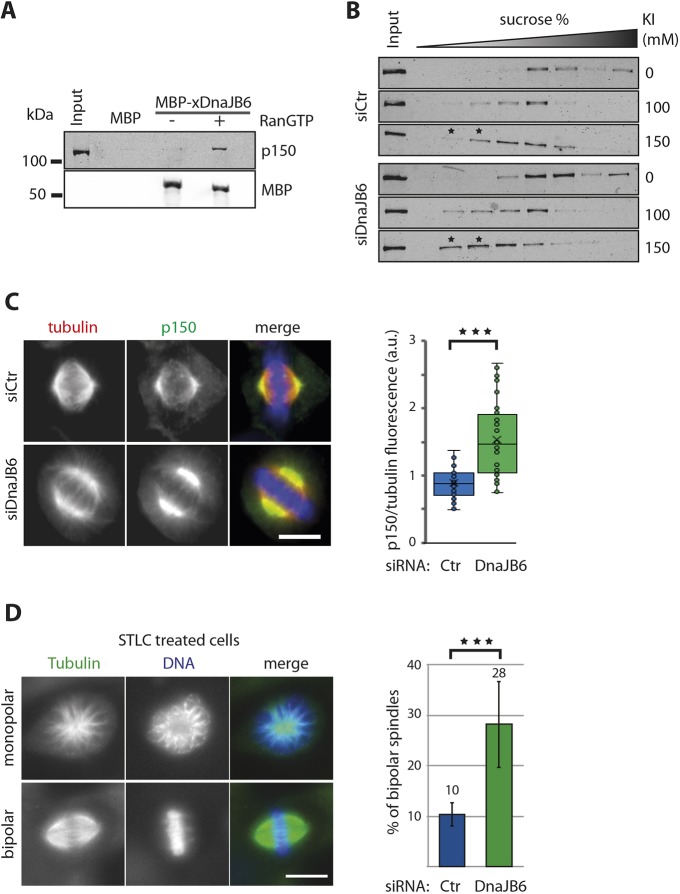
**DnaJB6 regulates dynactin spindle localization and is required for dynein-dependent force generation within the spindle.** (A) Western blot analysis of a MBP–xDnaJB6-L pulldown experiment in *Xenopus* egg extract. MBP–xDnaJB6-L- or MBP-coated Dynabeads were incubated in egg extracts in the presence or absence of RanGTP. p150Glued was specifically pulled down with MBP–xDnaJB6-L from extracts containing RanGTP. The lower panel shows that similar amounts of MBP–xDnaJB6-L was used for pulldown both in the presence and absence of RanGTP. (B) Western blot analysis showing the position of p150Glued in 8–20% sucrose density gradients from lysates of control and DnaJB6-silenced cells. Lysates were prepared from mitotic cells. KI was added to the lysates and incubated for 1 h before running the gradients, at the concentrations indicated on the right. A shift to in where p150Glued is detected in to fractions with a lower percentage of sucrose is observed in mitotic DnaJB6 cell lysates treated with 150 mM KI compared to the mitotic control cells lysates with the same treatment, as highlighted with asterisks. (C) p150Glued accumulates at spindle poles in DnaJB6-silenced cells. Left: representative immunofluorescence images from control and DnaJB6-silenced HeLa cells showing the localization of p150Glued in metaphase spindles. In the merge, tubulin is in red, dynein is in green and DNA is in blue. Scale bar: 10 μm. Right: box-and-whisker plot showing the value of the of p150Glued signal normalized to that of the tubulin signal (a.u., arbitrary units) in each spindle pole in control and DnaJB6-silenced HeLa cells. More than 30 cells were analyzed for each condition in one representative out of three independent experiments. (D) DnaJB6 silencing rescues spindle bipolarity in STLC-treated HeLa cells. Left: immunofluorescence images of representative monopolar and bipolar spindles in DnaJB6-silenced HeLa cells incubated with STLC. Tubulin is shown in green and DNA in blue. Scale bar: 10 μm. Right: bars graph showing the mean±s.d. percentage of bipolar spindles in control or DnaJB6-silenced HeLa cells incubated with STLC. Data from three independent experiments in which 937 control and 1057 DnaJB6-silenced cells were analyzed. ***P<0.001 (ANOVA test).

The defects in spindle organization that we observed upon DnaJB6 silencing in HeLa cells or depletion in *Xenopus* egg extracts (including spindle misorientation, centrosome detachment, spindle pole focusing defects and spindle length increase) are all compatible with altered dynein activity (Kardon and Vale, 2009; Raaijmakers and Medema, 2014). Pulldown experiments in egg extracts using MBP–xDnaJB6-L- or MBP-coated beads in the presence or absence of RanGTP showed that the dynactin subunit p150Glued was specifically pulled down by MBP–xDnaJB6 and only in the presence of RanGTP (Fig. 6A). Therefore DnaJB6 interacts with p150Glued in a RanGTP-dependent manner during M-phase.

Since DnaJB6 is a co-chaperon, we reasoned that its interaction with p150Glued could have a role in the assembly or stabilization of the dynactin complex. To test this idea, we performed sucrose density gradient analysis of cell lysates in the presence of the chaotropic agent potassium iodide (KI), which has previously been shown to promote the dissociation of the dynein complex (King et al., 2002). Control and DnaJB6-silenced HeLa cells were synchronized in interphase and mitosis, and the cell lysates incubated with KI before loading on top of an 8–20% sucrose density gradient. In control cells, western blot analysis showed that p150Glued migrated to lighter fractions of the gradient in the presence of KI. This indicated that KI induced the dissociation of the p150Glued complex both in interphase and mitosis, as described for the dynein complex. DnaJB6 silencing did not change the dissociation pattern of the p150Glued complex in interphase (Fig. S4). In contrast, it promoted a shift of p150Glued to lower sucrose concentrations in mitosis (Fig. 6B). These results suggest that DnaJB6 is required for the stability of a p150Glued complex specifically in mitosis. This complex may be the dynactin complex itself or a larger complex including dynein.

We then checked whether p150Glued spindle localization was affected in the absence of DnaJB6 both in egg extracts and in cells. Spindles assembled in egg extracts showed that p150Glued localized all along the spindle microtubules with some enrichment at the spindle poles. In the absence of DnaJB6, p150Glued accumulated at the spindle poles (Fig. S2A,B). This was not related to changes in p150Glued protein stability since there was no change in the general levels of p150Glued in control and DnaJB6-depleted extracts (Fig. S2C). Rescue experiments showed that p150Glued accumulation was reverted to control levels at the spindle poles upon addition of MBP–xDnaJB6-L to the depleted extract (Fig. S2A,B). These results indicate that DnaJB6 plays a role in p150Glued spindle pole localization in egg extracts.

Consistent with the above experiments, p150Glued also accumulated at the spindle poles in DnaJB6-silenced cells (Fig. 6C). We then checked dynein localization and found that the dynein intermediate chain and heavy chain (DIC and DHC, respectively) family proteins also localized more prominently to the spindle poles in DnaJB6-silenced cells. (Fig. S3B,C). Neither DIC proteins, DHC proteins nor p150Glued total protein levels were affected by the silencing of DnaJB6 in HeLa cells (Fig. S3A). Finally, we also found spindle pole enrichment of NuMA (Fig. S3D), recently shown to recruit p150Glued to the microtubule minus-ends in mitosis (Hueschen et al., 2017), although to a lesser extent than dynactin or dynein.

Taken together, our data indicates that DnaJB6 may be required for dynein function during mitosis possibly though an interaction with the dynactin complex in a RanGTP-dependent manner.

### DnaJB6 favors the generation of inward forces within the spindle

The dynactin complex is the major adaptor for dynein, promoting its processivity and force generation (Cianfrocco et al., 2015; Grotjahn et al., 2018; Jha and Surrey, 2015). The phenotypes associated with the absence of DnaJB6 during mitosis are consistent with those described for interference with dynein in mitosis, including spindle pole focusing defects and multipolarity (McKinley and Cheeseman, 2017; Raaijmakers and Medema, 2014). Moreover, our sucrose gradient results are compatible with DnaJB6 being required for the stability of the dynactin or dynein–dynactin complexes. This suggests that DnaJB6 silencing could impair dynein activity during mitosis. It has been previously shown that interfering with dynein activity rescues the formation of bipolar spindles in Eg5-deficient cells (Ferenz et al., 2009; Mitchison et al., 2005; Raaijmakers et al., 2013; Tanenbaum et al., 2008). We therefore asked whether DnaJB6 silencing could affect dynein force generation and rescue spindle bipolarity in HeLa cells treated with S-trityl-l-cysteine [STLC, and Eg5 inhibitor (Mayer et al., 1999)]. Control and DnaJB6-silenced HeLa cells were incubated with STLC for 2 h, fixed and processed for immunofluorescence. As expected, a majority of control cells had monopolar spindles (88%). Interestingly, DnaJB6-silenced cells had a significant increase in the proportion of cells with bipolar spindles to 28%, instead of 10% in the control cells (Fig. 6D). To test whether DnaJB6 could function through Hsp70 for inward force regulation, we tested whether a general HSP70 inhibitor (VER155008, Massey et al., 2010; Schlecht et al., 2013) could produce a similar bipolar spindle assembly rescue in STLC-treated cells. We did not observe any rescue by inhibiting HSP70, indicating a direct role for DnaJB6 in the regulation of inward force production during spindle assembly (Fig. S5A,B). The lower level of inward spindle forces suggested in DnaJB6-silenced cells treated with STLC is indeed consistent with the increase in spindle length in DnaJB6-silenced HeLa cells (Fig. 3A), a correlation also shown in previous publications (Morales-Mulia and Scholey, 2005; Goshima et al., 2005; Mitchison et al., 2005).

Taken together, our results suggest that DnaJB6 is required for dynein-dependent spindle inward force generation during mitosis.

## DISCUSSION

Here, we showed that the nuclear protein DnaJB6 interacts with dynactin p150Glued in a RanGTP-dependent way during mitosis by performing pulldown experiments. Moreover, all the phenotypes that we observed upon silencing or depleting DnaJB6 in cells and egg extracts are consistent with defects in dynein function. Our work therefore suggests a novel mechanism by which the small GTPase Ran, in its GTP-bound form, regulates the activity of the dynein–dynactin complex during M-phase to promote inward force production and spindle pole focusing.

Two isoforms of DnaJB6 are present in human cells but only the long isoform localizes to the nucleus in interphase and to the spindle in mitosis. DnaJB6 also interacts with p150Glued in a RanGTP-dependent manner in M-phase egg extracts. Silencing experiments reduced the levels of both the short and the long isoforms, but the destabilization of the dynactin complex only occurred in mitosis suggesting that the interphase cytoplasmic short isoform plays no role in dynactin stabilization and function. Moreover, our data show that the long isoform of DnaJB6 fully rescues the phenotypes of the DnaJB6 depletion in egg extracts and the distribution of p150Glued in the spindle. Taken together, these data strongly suggest that the function in spindle assembly that we describe here is specific for the long isoform of DnaJB6.

DnaJB6 is a member of the HSP40 protein family. The best-characterized role of several members of this family is to act as co-chaperons of HSP70 proteins. Some members of the HSP70 family were previously shown to play a role in spindle assembly (Fang et al., 2016; O'Regan et al., 2015). Here, we found that HSP70 inhibition does not rescue spindle bipolarity in Eg5-inhibited cells nor does it generate spindle pole focusing defects (data not shown). Moreover, the rescue experiments in egg extracts support a direct role for DnaJB6 in mitosis since the recombinant protein that rescued the depletion phenotype was added to the depleted extract only as the extract was sent into mitosis (Fig. 5; Fig. S2). Taken together, our data strongly suggest that the role of DnaJB6 in spindle assembly that we describe here is independent of HSP70. In agreement with this, HSP70-independent functions have been reported for some HSP40 family proteins including DnaJB6 (Hageman et al., 2010).

Dynein is a major minus-end-directed motor that performs a large number of functions in the cell. During mitosis it is essential for centrosome separation, chromosome alignment, spindle pole focusing, spindle positioning, centrosome coupling to spindle poles and mitotic checkpoint silencing, in addition to generating the inward forces that balance those mediated by Eg5 (Kardon and Vale, 2009; Raaijmakers and Medema, 2014; Goshima, 2005; di Pietro et al., 2016). All the phenotypes observed in DnaJB6-silenced cells are compatible with an impairment of dynein function: centrosome mispositioning, spindle misorientation, spindle pole focusing defects and an increase in spindle length. The increase in spindle multipolarity may also result from unbalanced forces within the spindle. Indeed a weakening of inward forces produced by dynein may result in dominant outward forces through Eg5, which could result in the disruption of the spindle pole integrity and the formation of multipolar spindles (Maiato and Logarinho, 2014). In agreement with this idea, we found that DnajB6 silencing restores spindle bipolarity when Eg5 is inhibited.

The wide range of dynein functions raises the question of how this motor is regulated. Several interacting proteins have been found to provide specificity for dynein localization and/or regulation (Cianfrocco et al., 2015). One of the best-characterized general regulators of dynein is dynactin, a large protein complex that participates in most dynein functions. Dynactin was shown to act as a dynein activator increasing its processivity and force production (Culver-Hanlon et al., 2006; Grotjahn et al., 2018; Kardon et al., 2009; King and Schroer, 2000; Ross et al., 2006; Tripathy et al., 2014).

We have shown that the long isoform of DnaJB6 pulls down the dynactin subunit p150Glued in a RanGTP-dependent manner during mitosis. This interaction may play a role in dynactin complex stability, as indicated by its reduced sensitivity to KI-induced disassembly in mitosis. However, and surprisingly, dynactin and dynein, as well as NuMA accumulate at the spindle poles in DnaJB6-silenced cells. These data are not very easy to reconcile, and additional work will be needed to understand precisely the mechanism by which DnaJB6 affects dynein function and localization during mitosis. However, our data overall point to an important role for DnaJB6 in modulating the functioning of dynactin and dynein during spindle assembly.

In summary, we describe here the identification and functional characterization of DnaJB6 as a novel RanGTP-regulated protein required for microtubule organization during mitosis, and suggest that it is important for dynein functions in the spindle.

## MATERIALS AND METHODS

### HeLa cell culture, DnaJB6 silencing and drug treatments

Cells were grown at 37°C in a 5% CO_2_ humid atmosphere. HeLa cells were grown in Dulbecco's modified Eagle medium (DMEM) with 4.5 g/l glucose and Ultraglutamine 1 (bioWhittaker, BE12-604F/U1), 10% fetal bovine serum (FBS; 102070-106, Invitrogen) and 100 units/ml penicillin and 100 µg/ml streptomycin (15140-122, Invitrogen). HeLa cells stably expressing H2B–eGFP and α-tubulin–mRFP were a gift from Patrick Meraldi (ETH, Zurich, Switzerland) (McAinsh et al., 2006) and were grown in the presence of 400 µg/ml G418 and 20 µg/ml puromycin. Cells were periodically tested for mycoplasma contamination.

Short interfering RNA (siRNA)-mediated interference oligonucleotides targeting DnaJB6 (5′-CUAUGAAGUUCUAGGCGUG-3′) or scrambled (5′-CGUACGCGGAAUACUUCGAUU-3′) were transfected into HeLa cells with Lipofectamine RNAmax (13778-150, Invitrogen) as detailed in the manufacturer's protocol; 4 μl of 20 μM siRNA were used for each well of a six-well plate. All experiments were performed after an incubation at 37°C for 48 h after transfection.

For microtubule regrowth experiments, cells were incubated for 3 h at 37°C with 2 μM nocodazole (Sigma, M1404). Nocodazole was then washed out by three washes with 2 ml of pre-warmed 1× PBS and one of medium at 37°C. Cells were incubated for different periods in a 37°C incubator, fixed in −20°C methanol for 10 min and processed for immunofluorescence using the DM1A antibody (see below).

For the spindle bipolarization rescue experiment, control and DnaJB6-silenced cells were incubated for 2 h at 37°C with 2 μM STLC (Sigma, 164739), fixed in −20°C methanol and processed for immunofluorescence using the DM1A or anti-tubulin-β antibodies.

### Cloning of *Xenopus laevis* DnaJB6

*Xenopus* mRNA was obtained from CSF *Xenopus leavis* egg extracts using TRIzol reagent (15596-026, Thermo Fisher Scientific) according to manufacturer's instructions. cDNA was generated by RT-PCR using an oligo-dT coupled with an adaptor sequence (5′-GGCCACGCGTCGACTAGTAC +17 ‘T’-3′). The 3′-end sequence of the mRNA was obtained by PCR using the Phusion High-Fidelity DNA Polymerase (F530S; Thermo Fisher Scientific) according to the manufacturer instructions, using a primer with the adaptor sequence and another corresponding to the central part of the xDnaJB6-S (NM_001095775.1) annotated sequence that is conserved between the long and the short isoforms in human (5′-GGAGGTTTCCCTGCCTTTGGCCC-3′). In a second step, another PCR was performed on the *Xenopus* egg cDNA using a reverse primer corresponding to the 3′-end of the long isoform previously obtained and a forward primer containing the annotated start codon from xDnaJB6-S (fwd 5′-ATGGTGGAGTATTACGAAGTTTTGGGAGTCC-3′; rev 5′-TTAGTAGATTGGTTTGGAAGACTTCTTTTTCTTG-3′). The sequence for the xDnaJB6 long isoform was deposited into the GenBank database (https://www.ncbi.nlm.nih.gov/nuccore/MK914533).

### Protein production

The full-length xDnaJB6-L sequence was cloned into the pGex-4T-2 (Amersham Pharmacia Biotech) and pMal-c2 (New England Biolabs) vectors. MBP–xDnaJB6 was expressed in the *E. coli* BL21/RIL strain by induction with 0.5 mM IPTG for 4 h at 25°C. The protein was purified on amylose resin beads (E8021S, NEB) according to the manufacturer’s instructions. The purified protein was stored in 20 mM Tris-HCl pH 7.4 with 200 mM NaCl at −80°C.

GST–xDnaJB6 was expressed in the *E. coli* BL21pLys strain by incubation with 1 mM IPTG overnight at 20°C. For protein purification, glutathione–Sepharose beads (17-5132-01, GE Healthcare) were used according to manufacturer's instructions. Protein was kept in 50 mM Tris-HCl pH 8.0 with 200 mM KCl at −80°C.

### Antibodies

Affinity-purified antibodies against human (h)DnaJB6 and GST were previously described (Rosas-Salvans et al., 2018). Antibodies against xDnaJB6 were obtained by rabbit immunization with MBP–xDnaJB6-L and affinity purified. The working concentrations were as follows: anti-hDnaJB6 antibody [immunofluorescence (IF), 5 μg/ml; WB, 1 μg/ml] and anti-xDnaJB6 antibody (WB, 1 μg/ml).

The following primary commercial antibodies were used: DM1A (Sigma, T6199; IF and WB, 1 μg/ml), anti-tubulin-β (Abcam, ab6046; IF, 1:200), anti-p150Glued (BD Transduction Laboratories, 610474; IF, 5 μg/ml; WB, 0.5 μg/ml), anti-centrin (Millipore, 04-1624; IF, 1:1000), anti-NuMA (Calbiochem, NA09L; IF, 1:500), anti-DIC (Millipore; MAB1618; IF, 1:500), anti-DHC (Santa Cruz Biotehcnology; SC514579; IF, 1:50), anti-Eg5 (BD Transduction Laboratories; 611186; IF, 1:1000), anti-importin-β/NTF97 (Abcam; ab36775-50; WB, 1:500) and generic IgGs (Sigma, I5006). The following secondary antibodies were used: Alexa Fluor 488-conjugated goat anti-mouse-IgG (A11017; Invitrogen), Alexa Fluor 488-conjugated goat anti-rabbit-IgG (A11034; Invitrogen), Alexa Fluor 568-conjugated anti-mouse-IgG (A11031; Invitrogen) and Alexa Fluor 568-conjugated goat anti-rabbit-IgG (A11036; Life Technologies) all at 0.2 μg/ml final concentration. For western blots, the following secondary antibodies were used: anti-mouse-IgG conjugated to IRDye 800 (926-32212; Licor), goat anti-rabbit-IgG conjugated to IRDye 800 (10733944; Thermo Fisher Scientific), goat anti-rabbit-IgG conjugated to Alexa Fluor 680 (A-21109; Invitrogen) and goat anti-mouse-IgG conjugated to Alexa Fluor 680 (A21058; Life Technologies) diluted at 1:10,000.

### *Xenopus* egg extracts

All the experiments involving animals were performed according to standard protocols approved by the ethical committee of the Parc de Recerca Biomèdica de Barcelona. *Xenopus laevis* frogs (males and females) were purchased from Nasco and used at an age between 1 and 3 years. CSF-arrested egg extracts were prepared and used to perform cycled spindle assembly assays as previously described (Desai et al., 1999).

For depletion experiments, protein A-conjugated Dynabeads 280 (10002D, Invitrogen) were coated with anti-xDnaJB6 antibody or generic IgGs (9 μg of antibody/30 μl of beads) and incubated in freshly prepared egg extract for 30 min on ice. Beads were recovered and protein depletion efficiency tested by western blot analysis. For add-back experiments, 0.017 μM (estimated endogenous concentration of DnaJB6 in the egg extract) of MBP–xDnaJB6-L or MBP were added to the depleted egg extracts. To look at the effects of increasing the concentration of DnaJB6, we added 1 μM of MBP or MBP–xDnaJB6-L to egg extracts.

### Pulldown experiments from egg extracts

Protein A-conjugated Dynabeads 280 (10002D, Invitrogen) were coated with anti-GST or anti-MBP antibody (9 μg of antibody/30 μl of beads), and incubated with recombinant proteins (GST, GST–xDNAJB6-L, MBP or MBP–xDnaJB6-L). Protein-coated beads were then incubated in CSF egg extract (30 μl of beads/75 μl of egg extract) for 15 min at 20°C followed by 30 min on ice. Recombinant RanQ69L-GTP was added at 15 μM. Beads were recovered and washed three times in CSF-XB (10 mM HEPES pH 7.7, 50 mM sucrose, 100 mM KCl, 0.1 mM CaCl_2_ and 5 mM EGTA; for checking importin interaction) or three times in CSF-XB and one in PBS containing NP40 (for checking p150Glued interaction). Proteins were eluted from the beads by incubation in sample buffer for 10 min at room temperature and run on SDS-PAGE before western blotting.

### Immunofluorescence

Cells grown on coverslips were fixed in −20°C methanol for 10 min. They were blocked and permeabilized for 30 min in IF-buffer (1× PBS, 0.1% Triton X-100 and 0.5% BSA). Primary antibodies diluted in IF-buffer were then applied for 1 h at room temperature. Samples were washed three times in IF-buffer and secondary antibodies and Hoechst (Hoechst 33342; ref: H3570; Invitrogen; added at 1:1000) were applied for 45 min. The coverslips were washed with IF-buffer and twice with PBS before mounting in Mowiol. For anti-DnaJB6 immunostaining, cells were pre-extracted for 6 s in 1× BRB80, 0.5% Triton X-100 and 1 mM dithiobis(succimidyl propionate) (DSP). Cells were then fixed in PFA 4% for 7 min and then processed for IF as described.

Spindles assembled in egg extracts were centrifuged onto coverslips as previously described, fixed in −20°C methanol for 10 min and processed for IF following the above protocol.

### Sucrose gradients

Sucrose gradient (8 to 20%) experiments were performed as described in (Jones et al., 2014). For preparing interphase cell lysates, cells were collected in 5 ml of PBS using a scraper. For preparing mitotic lysates, cells were incubated for 15 h in 2 μM nocodazole, washed 3× with PBS and once with medium (20 ml). They were then incubated at 37°C for 45 min when the majority of the cells were in metaphase. Cells were recovered by shake off. To prepare the lysates, cells were washed twice in 1× BRB80 and centrifuged at 600 ***g*** for 5 min. The pellets were resuspended in 500 μl of lysis buffer (1× BRB80, 0.5% Triton-X100 with protease inhibitors) for 15 min on ice and subsequently centrifuged at 16,100 ***g*** (in a 5415 R Eppendorf centrifuge) for 10 min at 4°C. The protein concentration of the supernatants was measured by performing a Bradford assay. 100 μl of control or DnaJB6-silenced cell lysates were incubated with KI (0, 100 or 150 mM) and incubated for 1 h on ice. 45 μl of each sample was added to the top of sucrose gradients in centrifuge tubes previously prepared at room temperature. Tubes were centrifuged at 38,000 rpm for 5 h at 4°C in a SW-55Ti rotor with an ultracentrifuge Beckman optima L-100K. 75 μl fractions were recovered from the top and diluted in sample buffer. Samples were then loaded on SDS-PAGE gels and subjected to western blotting.

Sucrose gradients were prepared in 800 µl (0.8 ml) UltraClear Centrifuge Tubes, 5×41 mm (part Number 344090) and specific adaptors used for centrifugation (Adapter, Split, Delrin, Tube, 5 mm diameter, qty. 2 halves, product number 356860). Sucrose solutions were prepared in BRB80 containing protease inhibitors (1:4000), 0.1 mM ATP, 1 mM DTT, sucrose and KI at the selected concentrations (0, 100 or 150 µM KI). The gradient was prepared by overlaying manually the different sucrose solutions (2% difference of sucrose between layers, seven layers from 20 to 8% of sucrose), freezing between each layer addition. The gradients were kept at −20°C and warmed up to room temperature for 1 h before use.

### Microscopy

Non-synchronized HeLa cells stably expressing H2B–eGFP and α-tubulin–mRFP were imaged every 4 min for 18 h with a 40× objective on a Zeiss Cell Observer HS inverted microscope equipped with a a Zeiss AxioCam MrX camera and temperature, humidity and CO_2_ control systems. Images were processed and analyzed with FIJI software.

Immunofluorescence images were obtained with an inverted DMI-6000-B Leica wide-field fluorescent microscope equipped with 40× and 63× objectives and a Leica DFC 360FX camera. Confocal images were obtained on a Leica TCS SPE microscope equipped with a 63× objective and the following laser lines: 405 nm (for DAPI and Hoechst), 488 nm (for GFP and FITC), 532 nm (for mRFP, DsRED, Cy3, TRITC and TexasRed), 635 nm (for Cy5 and DRAQ5).

### Microscopy imaging quantifications

Images taken with a 63× objective on an inverted DMI-6000-B Leica wide-field fluorescent microscope, equipped with a Leica DFC 360FX camera, were analyzed using the FIJI program as follows.

### Spindle profile quantifications

Spindles were individually rotated in order to orient them with a horizontal pole-to-pole axis. A rectangle of a fixed size containing both spindle poles and centered on the metaphase plate was drawn on each image. The average pixel intensity was measured for sequential vertical pixel lines, obtaining a list of intensities, which was exported to an independent file. The intensity lists for all the cells analyzed in any given condition were analyzed using the software Prism and plotted together. A line graph was generated with a line showing the average intensities in each vertical pixel line and the standard deviations. In order to compare the protein intensities at the spindle poles, the length of the spindles was normalized by subtracting the central spindle values of the larger spindles, when necessary. Protein intensities were normalized using the intensity of the tubulin (obtained by the same method). The graphs obtained for DnaJB6-silenced cells and control cells were compared with a one-way ANOVA test to obtain the significance of the differences between the two conditions.

### Quantification of fluorescence signal at spindle poles

Metaphase HeLa cells were randomly selected and subsequent measurements were made with ImageJ software. The tubulin signal was thresholded (by a rolling ball method with radius of 50 pixels; ImageJ threshold option), a selection on each mitotic spindle pole was generated and tubulin total intensity. On the same selection, the total fluorescence of the protein of interest was measured, corrected by means of background subtraction and finally normalized to the overall tubulin intensity.

#### Spindle poles width quantification

Metaphase HeLa cells were randomly selected. Centrosomes were labeled using an anti-centrin and anti-tubulin antibodies. The centrin signal was used to define the centrosome position. Subsequent measurements were made with ImageJ software. The tubulin signal was thresholded (by a rolling ball method with radius of 50 pixels; ImageJ threshold option) and the distance between the two closest spindle borders crossing the centrosome position was measured.

#### Quantification of centrosome misplacement

Centrosomes were labeled using an anti-centrin antibody. Metaphase HeLa cells were randomly selected. Metaphase plate axis was defined based on the aligned chromosomes. Subsequent measurements were made with ImageJ software. The centrosome axis was defined as a line connecting the two centrosomes. In control cells, the two lines had an angle close to 90°. A mark was also placed on the centrosome-to-centrosome mid-point that falls in close proximity to the metaphase plate axis in control cells. To quantify centrosome displacement in DnaJB6i cells the angle (α) between the centrosome and the metaphase plate axis was measured. To represent centrosome axis deviation, the α angle was subtracted from 90° and represented in a box-and-whisker plot. To quantify centrosome disengagement from the spindle, the distance between the centrosome-to-centrosome mid-point and the metaphase plate was measured and plotted in a box-and-whisker plot.

### Statistical analysis

PRISM software was used for the statistical analysis. Depending on the nature of the data, a one-way or two-way ANOVA test, a Mann–Whitney test, a Fisher's exact test or a Student's *t*-test was used. Specifically, one-way ANOVA was used in Fig. 6C, two-way ANOVA was used in the rest of the cases applying a Tukey's multiple comparisons test in Fig. 5C and a Sidak's multiple comparisons test in the rest of the cases. Normality distribution was tested (D'Agostino–Pearson omnibus normality test) for each sample when applicable, and equality of variances between samples in compared conditions was tested when necessary. In box-and-whisker plots, boxes show the upper and lower quartiles (25–75%) with a line at the median, whiskers extend from the 10–90th percentiles, and dots outside of whisker ranges correspond to outliers. The + indicates the mean values*.* Sample size was determined based on previous work done in the laboratory. For quantifications, the investigator was blind to the sample allocation at the moment of counting.

## Supplementary Material

Supplementary information
