# Supplementary material for: DnaJB6 is a RanGTP-regulated protein required for microtubule organization during mitosis
Source: J Cell Sci. 2019 Jun 3;132(11):jcs227033. doi: 10.1242/jcs.227033 (PMC6589090; doi:10.1242/jcs.227033)
Supplement: Supplementary information [file joces-132-227033-s1.pdf]

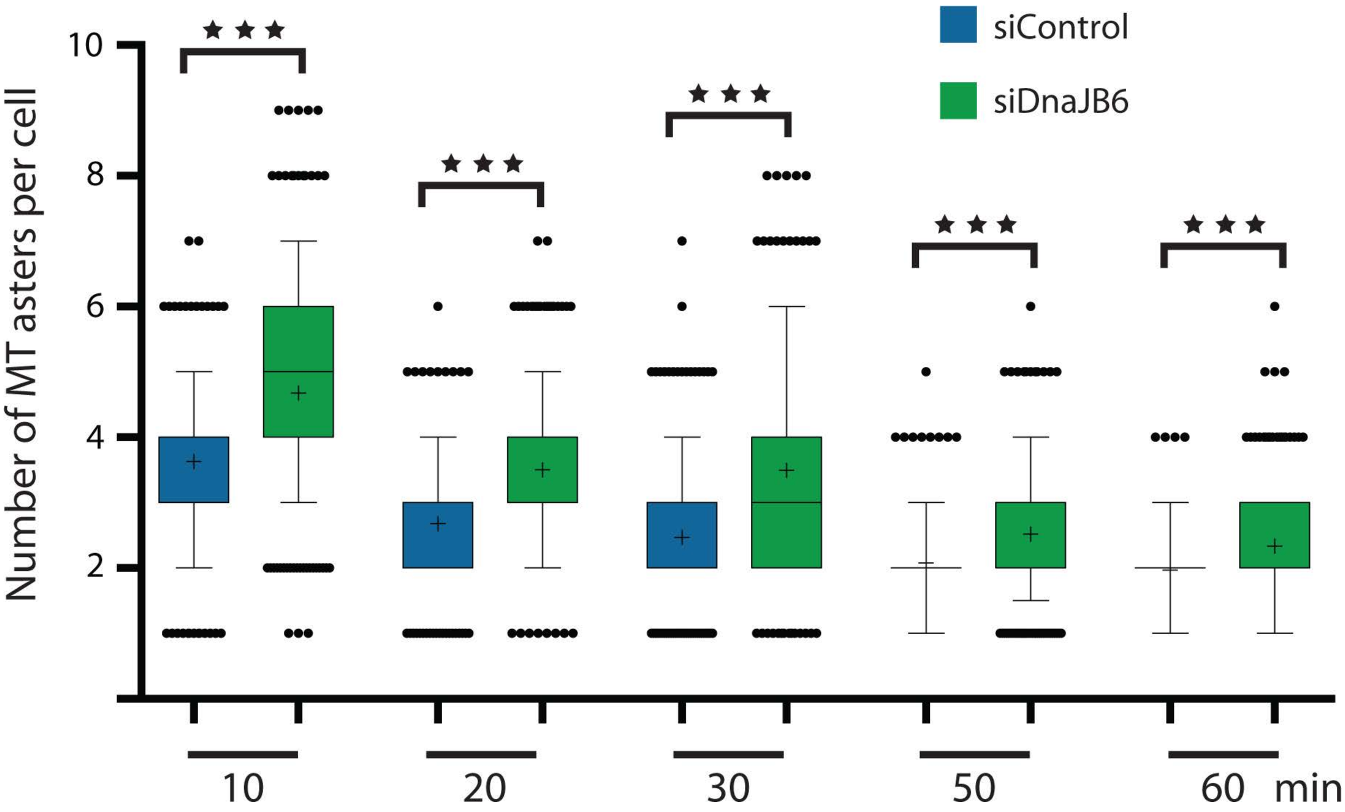

FIGURE S1

A) Box and whiskers plot of the number of microtubule asters quantified in mitotic control (blue) and DnaJB6 silenced (green) cells in a MT regrowth assay. Cells were fixed at the indicated time-points after nocodazole washout. Data from three independent experiments (total sample size: control cells: 350, 328, 310, 333, 317 control and DnaJB6 silenced cells: 353, 301, 330, 354 and 312). Three asterisks correspond to P-value<0.0001 (Mann-Whitney test).

Journal of Cell Science • Supplementary information

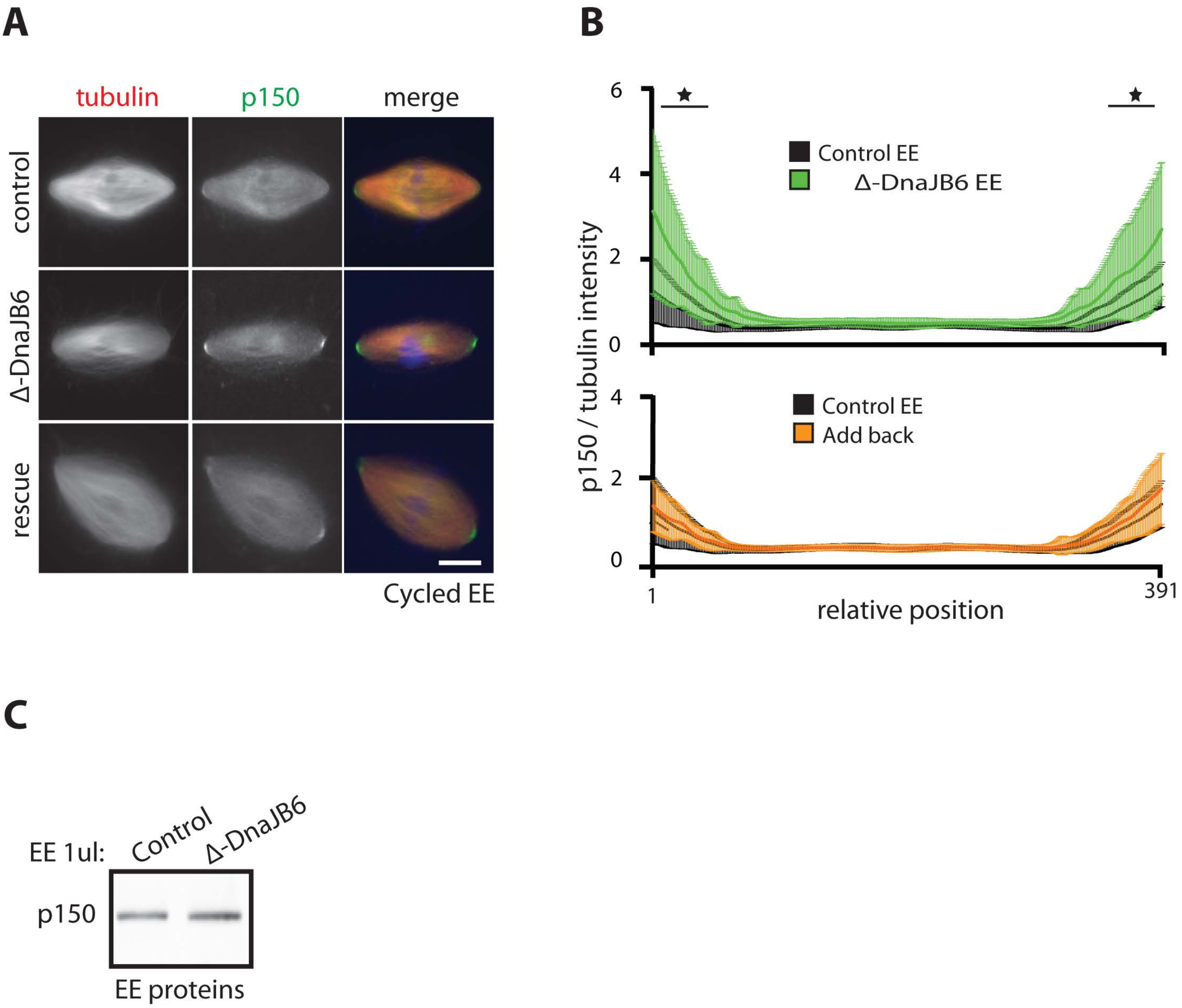

FIGURE S2

A) Immunofluorescence images showing P150Glued localization on bipolar spindles assembled in cycled control extracts, DnaJB6 depleted extracts or DnaJB6 depleted extracts supplemented with recombinant MBP-xDnaJB6-L. p150Glued is shown in green, tubulin in red and DNA in blue. Scale bar, 10µm.

B) Graphical representation of the p150Glued relative fluorescence signal intensity

Journal of Cell Science • Supplementary information

along the spindle. Fluorescence intensities from several images were analyzed using FIJI by drawing a rectangle (of a conserved size) from pole to pole on each spindle. The p150Glued intensity values were normalized using the tubulin fluorescence intensity signal. The average values are shown as a line within the standard deviation. Values from spindle assembled in control extracts are in black (n=20), those from spindles assembled in DnaJB6 depleted extracts are in green (n=16) and those assembled in DnaJB6 depleted extracts containing MBP-xDnaJB6-L (add back) are in orange (n=21). Metaphase spindles were selected randomly, excluding the spindles with pole focusing defects. A statistically significant accumulation of p150Glued at the spindle poles occurs in DnaJB6 depleted extracts ($P<0.05$, two tailed ANOVA test) and is rescued by addition of recombinant MBP-xDnaJB6-L to the depleted extract (lower graph).

C) Western blot analysis of control and DnaJB6 depleted egg extracts (1µl each) showing that the levels of p150Glued are similar.

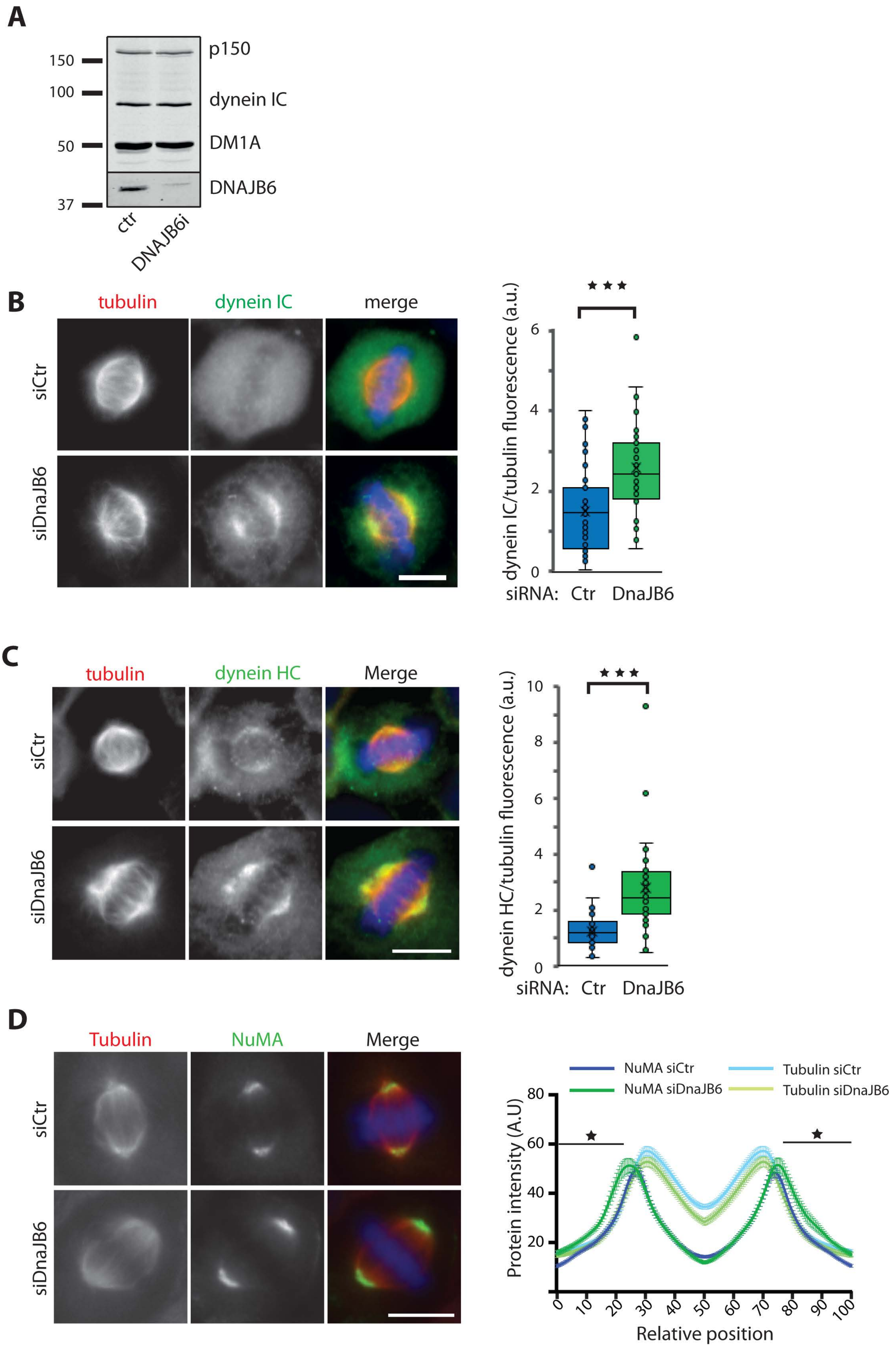
A
150
100
50
37
p150
dynein IC
DM1A
DNAJB6
ctr
DNAJB6i
B
tubulin
dynein IC
merge
siCtr
siDnaJB6
dynein IC/tubulin fluorescence (a.u.)
siRNA: Ctr DnaJB6
C
tubulin
dynein HC
Merge
siCtr
siDnaJB6
dynein HC/tubulin fluorescence (a.u.)
siRNA: Ctr DnaJB6
D
Tubulin
NuMA
Merge
siCtr
siDnaJB6
NuMA siCtr
NuMA siDnaJB6
Tubulin siCtr
Tubulin siDnaJB6
Protein intensity (A.U)
Relative position

FIGURE S3

A) Western blot analysis of mitotic HeLa cell extracts showing that p150Glued and Dynein intermediate chain (IC) levels are not affected by DnaJB6 silencing.

B) Dynein intermediate chain (IC) accumulates at spindle poles in DnaJB6 silenced cells. Left: representative immunofluorescence images from control and DnaJB6 silenced HeLa cells showing the localization of dynein intermediate chain in metaphase spindles. In the merge, tubulin is in red, dynein in green and DNA in blue. Scale bar 10µm. Right: box and whiskers plot showing measurements of dynein IC signal normalized with tubulin signal in each spindle pole in control and DnaJB6 silenced HeLa cells. More than 30 cells were analyzed for each condition in one representative out of three independent experiments. Three asterisks correspond to p-value<0.001 (Student t-test).

C) Dynein heavy chain (HC) accumulates at spindle poles in DnaJB6 silenced cells.

Left: representative immunofluorescence images from control and DnaJB6 silenced HeLa cells showing the localization of Dynein heavy chain in metaphase spindles. In the merge, tubulin is in red, dynein in green and DNA in blue. Scale bar 10µm. Right: box and whiskers plot showing measures of Dynein HC signal normalized with tubulin signal in each spindle pole in control and DnaJB6 silenced HeLa cells. More than 30 cells analyzed for each condition in one representative out of three independent experiments. Three asterisks correspond to p-value<0.001 (Student t-test).

D) NuMA accumulates at spindle poles in DnaJB6 silenced cells.

Left: representative immunofluorescence images from control and DnaJB6 silenced HeLa cells showing the localization NuMA in metaphase spindles. In the merge, tubulin is in red, NuMA in green and DNA in blue. Scale bar 10µm. Right: quantification of NuMA and tubulin fluorescence intensities measured along the spindle in control (blue) and DnaJB6 silenced HeLa cells (green). Protein fluorescence intensities were analyzed using FIJI. The relative positions along the spindle are indicated with arbitrary units. Mean values and the standard error of the mean are shown. NuMA fluorescence intensity is significantly increased at the spindle poles in DnaJB6 silenced cells. The significance was calculated after normalization of the p150Glued signal on the tubulin signal (P<0.05, two tailed ANOVA test). The measurements were obtained from 56 (control) and 36 (DnaJB6 silenced) metaphase spindles.

Journal of Cell Science • Supplementary information

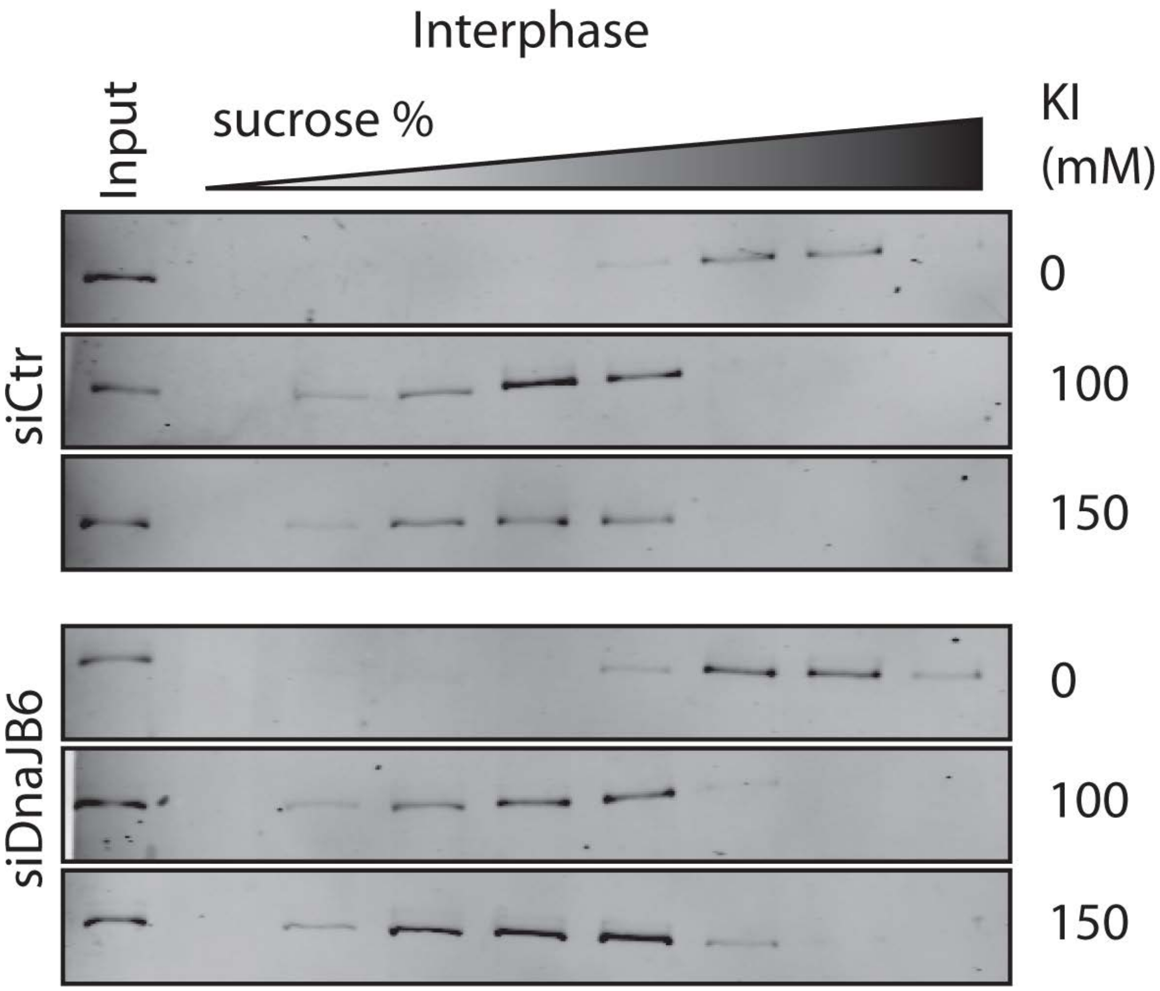

FIGURE S4

A) Western blot analysis showing the position of p150Glued in 8-20% sucrose density gradients from lysates of control and DnaJB6 silenced cells. Lysates were obtained form interphase HeLa cells. KI was added to the lysates for one hour before running the gradients, at the concentrations indicated on the left. No differences are observed between control and DnaJB6 silenced samples in interphase.

Journal of Cell Science • Supplementary information

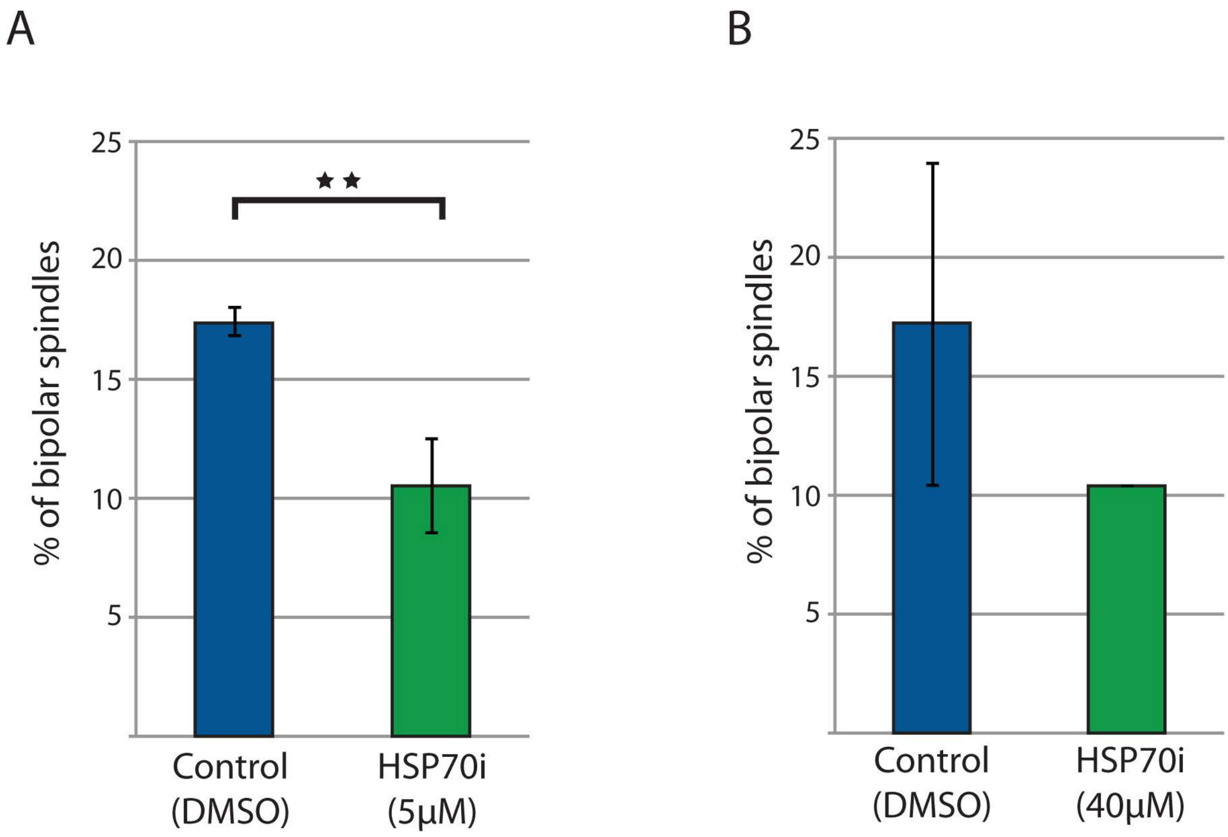

FIGURE S5

A) Bars graph showing the percentage of bipolar spindles in control and HSP70 inhibited HeLa cells treated with STLC. Cells were incubated with the HSP70 inhibitor Ver155008 at 5µM final concentration. A significant reduction of the percentage of bipolar spindles was detected in HSP70 inhibited cells. Data from two independent experiments monitoring 544 control and 549 HSP70 inhibited cells. Error bars represent standard deviation. Two asterisks correspond to p-value<0.01 (two tailed ANOVA test).

B) Graph showing the percentage of bipolar spindles in control and HSP70 inhibited HeLa cells treated with STLC. Cells were incubated with the HSP70 inhibitor Ver155008 at 40µM. Data from two independent experiments monitoring 450 control and 472 HSP70 inhibited cells. Error bars represent standard deviation. No significant differences were detected between the two conditions (two tailed ANOVA test).
